# The selective serotonin reuptake inhibitor fluoxetine has direct effects on beta cells, promoting insulin secretion and increasing beta‐cell mass

**DOI:** 10.1111/dom.14791

**Published:** 2022-07-11

**Authors:** Bo Liu, Inmaculada Ruz‐Maldonado, Klaudia Toczyska, Oladapo E. Olaniru, Mohammed Gulrez Zariwala, David Hopkins, Min Zhao, Shanta J. Persaud

**Affiliations:** ^1^ Department of Diabetes, School of Cardiovascular and Metabolic Medicine and Sciences, Faculty of Life Sciences & Medicine King's College London London UK; ^2^ Comparative Medicine and Pathology, Vascular Biology and Therapeutics Program (VBT) Program in Integrative Cell Signaling and Neurobiology of Metabolism (ICSNM) Yale University School of Medicine New Haven Connecticut USA; ^3^ Centre for Nutraceuticals, School of Life Sciences University of Westminster London UK

## Abstract

**Aim:**

This study investigated whether therapeutically relevant concentrations of fluoxetine, which have been shown to reduce plasma glucose and glycated haemoglobin independent of changes in food intake and body weight, regulate beta‐cell function and improve glucose homeostasis.

**Methods:**

Cell viability, insulin secretion, beta‐cell proliferation and apoptosis were assessed after exposure of MIN6 beta cells or isolated mouse and human islets to 0.1, 1 or 10 μmol/L fluoxetine. The effect of fluoxetine (10 mg/kg body weight) administration on glucose homeostasis and islet function was also examined in *ob/ob* mice.

**Results:**

Exposure of MIN6 cells and mouse islets to 0.1 and 1 μmol/L fluoxetine for 72 hours did not compromise cell viability but 10 μmol/L fluoxetine significantly increased Trypan blue uptake. The dose of 1 μmol/L fluoxetine significantly increased beta‐cell proliferation and protected islet cells from cytokine‐induced apoptosis. In addition, 1 μmol/L fluoxetine induced rapid and reversible potentiation of glucose‐stimulated insulin secretion from islets isolated from mice, and from lean and obese human donors. Finally, intraperitoneal administration of fluoxetine to *ob/ob* mice over 14 days improved glucose tolerance and resulted in significant increases in beta‐cell proliferation and enhanced insulin secretory capacity.

**Conclusions:**

These data are consistent with a role for fluoxetine in regulating glucose homeostasis through direct effects on beta cells. Fluoxetine thus demonstrates promise as a preferential antidepressant for patients with concomitant occurrence of depression and diabetes.

## 1 INTRODUCTION

Fluoxetine, the active pharmaceutical ingredient of the antidepressant Prozac®, is a selective serotonin reuptake inhibitor (SSRI) that is widely believed to mediate its antidepressant effects by enhancing serotonin (5‐hydroxytryptamine [5‐HT]) neurotransmission. When released, 5‐HT is rapidly retrieved following reuptake by the 5‐HT transporter, SERT.^1^ Fluoxetine binds to SERT directly to block the reuptake of 5‐HT from the synaptic cleft, leaving elevated levels of 5‐HT available to activate postsynaptic 5‐HT receptors and triggering downstream intracellular signalling cascades that reduce the symptoms of depression.^2^, ^3^, ^4^, ^5^

A reciprocal relationship between depression and type 2 diabetes (T2D) has long been reported, with depression being associated with a 60% increase in the risk of developing T2D^6^ and T2D being associated with a 24% increase in the risk of depressive symptoms.^7^ It is not clear whether a common aetiology accounts for the concomitant occurrence of these conditions, but there has been concern regarding increased risk of T2D with long‐term use of antidepressants.^8^, ^9^ However, a review of published trials concluded that it is the severe depression rather than the antidepressants that contributes to the risk of T2D, and SSRIs such as fluoxetine are the preferred choice of antidepressant to prevent long‐term metabolic risks.^10^ As such, use of fluoxetine in people with diabetes has resulted in reductions in both plasma glucose and glycated haemoglobin (HbA1c) levels^11^, ^12^, ^13^ and these effects are not influenced by depression status or changes in body weight.^14^ The mechanisms underlying improved glucose regulation in people taking SSRIs have not been established, but it is possible that these drugs are acting directly on insulin‐secreting beta cells to improve insulin secretion and/or expand beta‐cell mass.

5‐HT is synthesized de novo in beta cells via the rate‐limiting enzymes tryptophan hydroxylase 1 and 2 (Tph1 and Tph2)^15^ and aromatic‐L‐amino acid decarboxylase (AADC),^16^, ^17^ and it is stored in beta‐cell secretory vesicles.^17^ The importance of 5‐HT in glycaemic regulation is indicated by the impaired glucose homeostasis observed in mice deficient in peripheral 5‐HT.^18^ In addition, 5‐HT has been implicated in beta‐cell adaptive responses to pregnancy, with reports of enhanced insulin secretion and increased beta‐cell mass to compensate for peripheral insulin resistance.^15^, ^19^, ^20^, ^21^ It is thus possible that fluoxetine, by increasing local 5‐HT levels, has direct effects on the endocrine pancreas. Indeed, there have been earlier studies investigating the effects of SSRIs on beta cells, although these are of limited clinical relevance since fluoxetine was used at concentrations up to 100 μmol/L,^22^, ^23^, ^24^ which is considerably greater than the steady‐state plasma concentrations of 0.3 to 2.6 μmol/L detected in patients with chronic use of fluoxetine.^25^ The aim of this study, therefore, was to determine whether therapeutically relevant concentrations of fluoxetine have the potential to promote insulin release and/or beta‐cell expansion, both of which could have therapeutic benefits in diabetes.

## 2 MATERIALS AND METHODS

### 2.1 Materials

Fluoxetine hydrochloride, collagenase type XI, histopaque‐1077, 5‐bromo‐2′‐deoxyuridine (BrdU) and mouse monoclonal anti‐BrdU antibody were purchased from Sigma‐Aldrich (Poole, UK). Enhanced chemiluminescence (ECL) Western blotting detection reagents, Hyperfilm and Rainbow™ molecular weight markers were obtained from GE Healthcare (Amersham, UK). RNeasy mini kits, RNase‐free DNase sets and QuantiTect Primer Assays were from Qiagen (Manchester, UK). The high‐capacity cDNA reverse transcription kit was from Thermo Fisher Scientific (Paisley, UK). Anti‐SERT antibody was purchased from Abcam (Cambridge, UK), Anti‐phospho‐ERK, anti‐phospho‐AKT and anti‐phospho‐CREB primary antibodies were obtained from Cell Signaling (Hitchin, UK) and the anti‐insulin antibody used for immunohistochemistry was from Dako UK Ltd (Ely, UK). AlexaFluor 488‐ and AlexaFluor 594‐conjugated secondary antibodies were obtained from Jackson ImmunoResearch Laboratories (Newmarket, UK). Caspase‐Glo 3/7 and CellTiter‐Glo assay kits were from Promega (Southampton, UK). Recombinant murine tumour necro‐sis factor α (TNFα), interferon γ (IFNγ) and interleukin‐1β (IL‐1β) were from PeproTech EC (London, UK). PCR was carried out using a Px2 Thermal RT‐PCR cycler (Thermo Fisher Scientific, Paisley, UK). Accu‐Chek blood glucose meters and strips were from Roche Diagnostics (Burgess Hill, UK). MIN6 beta cells were kindly provided by Junichi I. Miyazaki (University of Osaka, Osaka, Japan).

### 2.2 Experimental animals

Male CD‐1, C57BL/6 and *ob/ob* mice (Envigo, Bicester, UK) were maintained at King's College London, with food and water supplied ad libitum. All animal procedures were approved by the King's College London Ethics Committee and carried out under licence, in accordance with the UK Home Office Animals (Scientific Procedures) Act 1986.

### 2.3 Isolation of mouse and human islets

Islets of Langerhans were isolated from 8‐week‐old male C57BL/6 mice by collagenase digestion, as described previously.^26^ Human islets of Langerhans were aseptically isolated from pancreases from nondiabetic, heart‐beating cadaver organ donors at King's College Hospital Islet Transplantation Unit with appropriate ethical approval.^27^

### 2.4 Trypan blue uptake

MIN6 beta cells were incubated with 0.1, 1 or 10 μmol/L fluoxetine or vehicle (0.005% v/v DMSO) for 72 hours. The cell membrane integrity of adherent cells was assessed by incubation in a Trypan blue solution (0.1% w/v) for 15 minutes. Cells in the wells were visualized on a Nikon TMS phase contrast microscope and photographs were obtained using a Canon EOS 4000D camera (Tokyo, Japan). In parallel experiments, MIN6 cells were incubated with 0.1, 1 or 10 μmol/L fluoxetine or vehicle (0.005% v/v DMSO) for 72 hours before being trypsinized. Cells to which the dye had gained access (blue) and nonstained cells (white) were then counted on a haemocytometer and percentage viable cells were calculated as percentage of nonstained cells over total number of cells. Viability of isolated mouse islets was assessed similarly except that islet cells were visualized on an Olympus KL300 LED microscope.

### 2.5 Cell proliferation

The effect of fluoxetine on MIN6 beta‐cell proliferation was quantified using a BrdU ELISA kit, as described previously.^28^ For the measurement of beta‐cell proliferation in islets, groups of 250 islets from CD‐1 mice were incubated with 1 μmol/L fluoxetine or vehicle (0.005% v/v DMSO) in the presence of 1 mg/mL BrdU for 5 days before being fixed and embedded in paraffin. Sections of 5‐μm thickness were treated with citrate buffer (10 mmol/L citric acid, 0.05% Tween 20, pH 6.0) and then immunostained with antibodies against BrdU and insulin at 1:100 and 1:200 dilutions, respectively. Immunopositive cells were detected using secondary antibodies conjugated to AlexaFluor 488 (BrdU; green) or AlexaFluor 594 (insulin; red) both at 1:50 dilution. Immunostained sections were visualized using a Nikon Eclipse TE2000‐U microscope (Tokyo, Japan), images were acquired with a Nikon Digital Sight‐Qi1Mc camera (Tokyo, Japan) and analysed using Image J software.

### 2.6 Apoptosis

Isolated mouse islets were maintained in culture for 72 hours in the presence of 0.1 or 1 μmol/L fluoxetine, or vehicle (0.005% v/v DMSO). For the last 24 hours of the incubation, a cytokine cocktail (0.025 U/μL IL‐1β, 1 U/μL TNFα and 1 U/μL IFNγ) was added to the culture medium. Groups of three islets were then transferred to white‐walled 96‐well plates and caspase 3/7‐induced cleavage of a luminogenic substrate was quantified using a luminometer as described previously.^29^

### 2.7 Static insulin secretion

Groups of three mouse and five human islets, randomized in size between treatment groups, were incubated with DMEM containing 2 mmol/L glucose for 1 h before being exposed to 2 mmol/L glucose DMEM or 20 mmol/L glucose DMEM in the absence or presence of 0.1 and 1 μmol/L fluoxetine for another hour. For chronic exposure experiments, isolated mouse or human islets were incubated in DMEM containing 2 or 20 mmol/L glucose in the absence or presence of 0.1 and 1 μmol/L fluoxetine for 72 hours. Islets were then incubated for 1 hour with 2 mmol/L glucose DMEM before being exposed to 2 or 20 mmol/L glucose DMEM for another hour. Insulin secreted into the supernatant was quantified by radioimmunoassay.^26^

### 2.8 Dynamic insulin secretion

Groups of 100 randomly selected islets, isolated from C57BL/6, *ob/ob* mice or lean (body mass index [BMI] 20 kg/m^2^) and obese (BMI 36 kg/m^2^) human donors, were transferred to chambers containing 1‐μm pore‐size nylon filters and perifused at 37°C and a flow rate of 0.5 mL/min in a physiological salt solution,^30^ under the conditions described for individual experiments. Samples were collected at 2‐minute intervals and secreted insulin was measured by radioimmunoassay.^26^

### 2.9 In vivo effects of fluoxetine

Groups of 29‐week‐old male *ob/ob* mice were administered four doses of fluoxetine (10 mg/kg body weight delivered in a volume of 0.4 mL DMSO/kg body weight) or vehicle (0.4 mL DMSO/kg body weight) intraperitoneally over 14 days before being subjected to intraperitoneal glucose tolerance tests (ipGTTs) or insulin tolerance tests (ipITTs), during which mice that were fasted overnight (for ipGTTs) or for 6 hours (for ipITTs) received a single intraperitoneal administration of glucose (2 g/kg body weight) or insulin (0.75 U/kg body weight) in the presence of fluoxetine (10 mg/kg body weight) or vehicle (0.4 mL/kg body weight DMSO). Tail vein blood glucose concentrations were determined with an Accu‐Chek blood glucose meter. BrdU (1 mg/mL) was delivered to mice in their drinking water for the last 7 days before the mice were killed. Pancreases were dissected from these mice, and BrdU incorporation into beta cells was quantified using immunohistochemistry as described above.

### 2.10 Reverse‐transcription PCR and quantitative real‐time PCR

RNA was extracted from MIN6 beta cells, mouse islets and human islets using RNeasy kits according to the manufacturer's protocol and reverse‐transcribed into cDNAs using a high‐capacity cDNA reverse transcription kit. cDNAs were then amplified over 40 cycles using prevalidated primers specific for mouse SERT (Mm_Slc6a4_1_SG QuantiTect Primer Assay QT00163345) and human SERT (Hs_SLC6A4_1_SG QuantiTect Primer Assay QT00058380). For quantification of SERT mRNA expression in islets from lean and obese donors, quantitative real‐time PCR was performed in a LightCycler 96 system using QuantiTech Primer Assays and SYBR Green Master I. Primer efficiency for SERT and glyceraldehyde‐3‐phosphate dehydrogenase (GAPDH) were 2.02 and 1.98, respectively. Template cDNA from three lean (BMI 21.0 ± 1.5 kg/m^2^) and three obese (BMI 31.3 ± 0.3 kg/m^2^) donors were diluted in such a way that all quantified genes returned cycle threshold values <26. Relative expression of SERT mRNA was determined after normalization against GAPDH mRNA expression in the same samples and calculated by the 2^−ΔΔCt^ method.^29^

### 2.11 Single‐cell calcium microfluorimetry

Islets isolated from three CD‐1 mice were dispersed using cell dissociation solution, and groups of 100 000 islet cells were seeded onto acid ethanol‐washed glass coverslips. Cells were maintained in culture overnight, and then loaded with 5 μM Fura‐2 AM and perifused (1 mL/min) on a heated stage with a physiological salt solution^30^ containing 20 mmol/L glucose in the presence of 1 μmol/L fluoxetine. Real‐time changes in [Ca^2+^]_i_ were determined by illuminating cells alternately at 340 and 380 nm, with the emitted light being filtered at 510 nm, and data were recorded with a charged‐coupled device camera every 3 seconds.

### 2.12 Western blotting

For the detection of SERT, 50‐μg protein extracts from MIN6 beta cells, human and mouse islets were subjected to polyacrylamide gel electrophoresis and Western blot analysis using an antibody against SERT (1:1000 dilution). For detection of ERK, Akt and CREB phosphorylation in response to fluoxetine, MIN6 beta cells were incubated with 1 μmol/L fluoxetine (delivered in 0.005% vol./vol. DMSO) or vehicle (0.005% vol./vol. DMSO) for 24 hours and parallel groups of cells were also exposed to 10 μmol/L forskolin or 100 nmoL/L insulin for 10 minutes. Cells were then lysed in the presence of phosphatase and protease inhibitors before being probed with antibodies against phospho‐ERK (1:1000 dilution), phospho‐Akt (1:1000 dilution), phospho‐CREB (1:1000 dilution) or beta actin (1:5000 dilution). Immunoreactivity was detected using X‐ray film after the addition of ECL substrate. Where necessary, antibodies were removed from polyvinylidene difluoride membranes by incubation with a phosphate‐buffered saline solution containing 2‐mercaptoethanol (0.7% vol./vol.) and sodium dodecyl sulphate (2% wt./vol.).

### 2.13 Statistical analyses

All statistical comparisons were made using Student's *t* tests or ANOVA, as appropriate. *P* values < 0.05 were taken to indicate statistical significance.

## 3 RESULTS

### 3.1 Therapeutic concentrations of fluoxetine do not compromise beta‐cell viability

**FIGURE 1:**
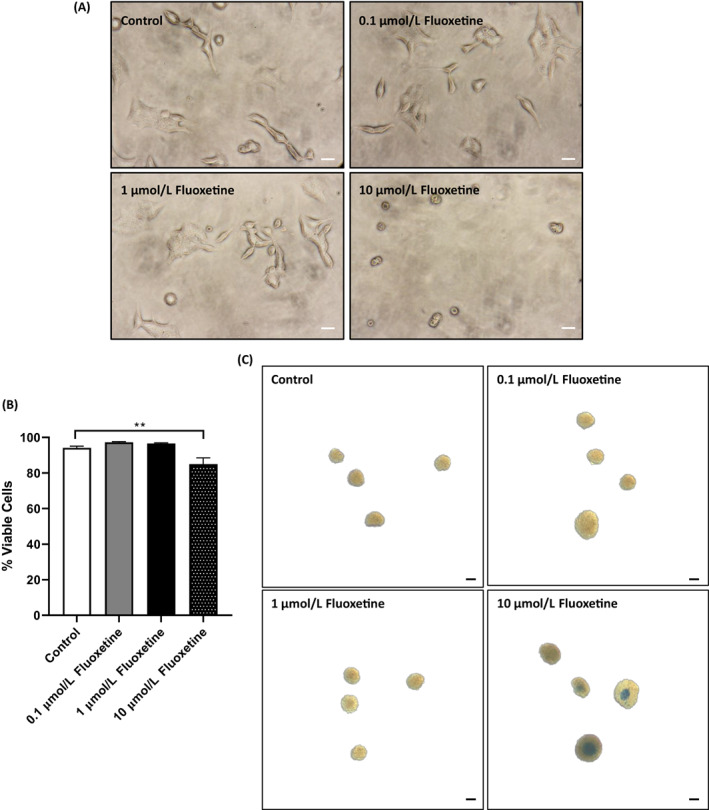
Effects of fluoxetine on beta‐cell viability. MIN6 beta cells growing in 96‐well plates were exposed to increasing concentrations of fluoxetine for 72 hours and viability of adherent beta cells was assessed by visualization of Trypan blue uptake (A, scale bars: 50 μm). In parallel experiments, MIN6 cells that had been exposed to increasing concentrations of fluoxetine for 72 hours were trypsinized and Trypan blue‐stained (blue) and nonstained (white) cells were quantified using a haemocytometer (B, *n* = 6, ****P < 0.0001 vs. control). For the measurement of islet cell viability, islets isolated from three CD‐1 mice were incubated with increasing concentrations of fluoxetine for 72 hours and Trypan blue uptake was assessed by light microscopy (C, scale bars: 100 μm). Trypan blue micrographs are representative of n = 10 replicates for MIN6 cells in three independent experiments and n = 6 replicates for mouse islets in three independent experiments

To examine the role of fluoxetine in regulating beta‐cell function, its effect on beta‐cell viability at both therapeutically relevant concentrations (0.1 and 1 μmol/L) and at a supra‐pharmacological concentration (10 μmol/L) were first measured. Almost all cells exposed to 0.1 and 1 μmol/L fluoxetine excluded entry of Trypan blue and their morphological characteristics were similar to those exposed to vehicle. However, 10 μmol/L fluoxetine caused significant increases in Trypan blue uptake (Figure 1A) and the cells developed a rounded appearance. Quantification of stained and nonstained cells demonstrated that 0.1 and 1 μmol/L fluoxetine did not reduce MIN6 cell viability whereas 10 μmol/L fluoxetine caused a 19.4 ± 2.3% decrease in the percentage of viable cells over the 72‐hour incubation period (*P* < 0.0001 vs. control; Figure 1B). Mouse islets exposed for 72 hours to 0.1 and 1 μmol/L fluoxetine also showed limited Trypan blue uptake while 10 μmol/L fluoxetine caused increased Trypan blue staining, particularly by peripheral cells of the islet apex (Figure 1C). These data indicate that therapeutic concentrations of fluoxetine do not compromise beta‐cell viability and fluoxetine was therefore used at 0.1 and 1 μmol/L for further experiments examining its role in regulating beta‐cell function.

### 3.2 Fluoxetine increases beta‐cell proliferation and decreases islet cell apoptosis in vitro

**FIGURE 2:**
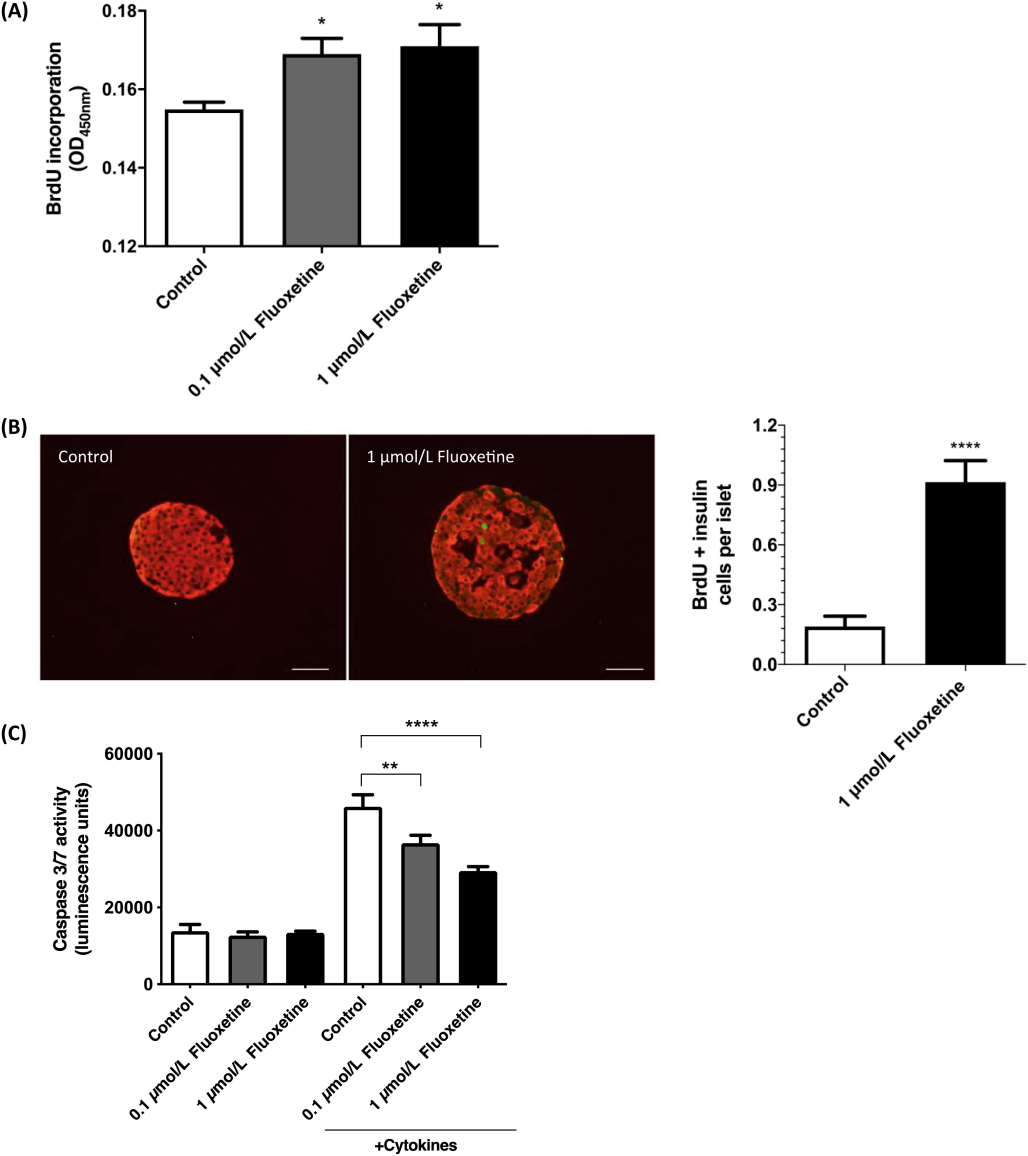
Effects of fluoxetine on beta‐cell proliferation and apoptosis. MIN6 beta cells were exposed to fluoxetine (0.1 and 1 μmol/L) for 72 hours, during which 10 μmol/L 5‐bromo‐2′‐deoxyuridine (BrdU) was present for the final 2 hours of incubation and beta‐cell proliferation was determined by quantifying BrdU incorporation into replicating DNA (A, n = 10, *P < 0.05 vs. control). Islets isolated from three CD‐1 mice were incubated with 1 μmol/L fluoxetine or vehicle (0.005% v/v DMSO) in the presence of 1 mg/mL BrdU for 5 days and wax‐embedded sections of islets were immunostained with antibodies directed against BrdU (green) and insulin (red; B, left panel, scale bars: 50 μm). The number of BrdU‐positive beta cells per islet was quantified by analysis of multiple acquisitions of 84 to 129 islets per condition, each with 12 paraffin sections (B, right panel, ****P < 0.0001 vs. control). Mouse islets were exposed to fluoxetine (0.1 and 1 μmol/L) for 72 hours, of which the final 24 hours were in the absence or presence of a cytokine cocktail, and islet cell apoptosis was determined by measuring caspase 3/7 activities (C, n = 8, representative of three independent experiments, **P < 0.01; ****P < 0.0001 vs. control)

Exposure of MIN6 cells to 0.1 and 1 μmol/L fluoxetine significantly increased BrdU incorporation (Figure 2A; *P* < 0.05) and this proliferative effect was also observed in isolated mouse islets where 1 μmol/L fluoxetine induced a significant increase in the number of insulin‐positive cells expressing BrdU (Figure 2B). Incubation of mouse islets with 0.1 or 1 μmol/L fluoxetine also significantly decreased cytokine‐induced islet cell apoptosis (Figure 2C), with the highest reduction in caspase 3/7 activities observed at 1 μmol/L.

### 3.3 Fluoxetine potentiates glucose‐induced insulin secretion in vitro

**FIGURE 3:**
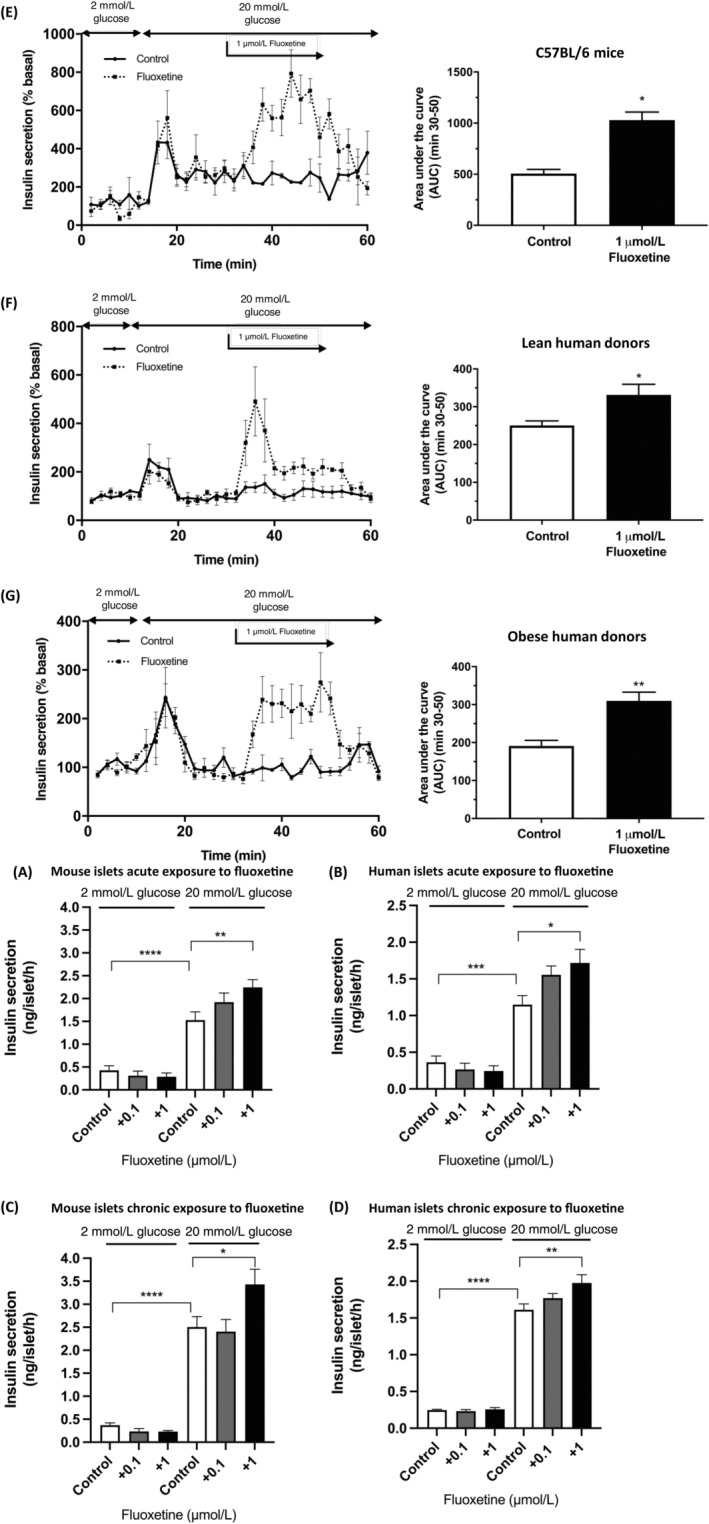
Effect of fluoxetine on insulin secretion in vitro. Groups of three islets, isolated from three CD‐1 mice (A), or five islets from human donors (B), were pre‐incubated with DMEM containing 2 mmol/L glucose for 1 hour before being exposed to medium supplemented with 2 mmol/L or 20 mmol/L glucose DMEM in the absence or presence of fluoxetine for another hour. Insulin secreted into the supernatant was quantified by radioimmunoassay. For chronic exposure expriments, isolated mouse (C) or human islets (D) were incubated in DMEM containing 2 or 20 mmol/L glucose and in the absence or presence of fluoxetine for 72 hours. Groups of three (mouse) or five (human) islets were then incubated for 1 hour with either 2 or 20 mmol/L glucose, in the absence of fluoxetine, and insulin secretion was measured using a radioimmunoassay. All data shown are mean + SEM, n = 9; one‐way ANOVA, ****P < 0.0001, **P < 0.1; *P < 0.05 relative to the control samples at 2 or 20 mmol/L glucose DMEM. Islets isolated from C57BL/6 mice (E), lean (F) and obese (G) human donors were perifused at 0.5 mL/min in a physiological salt solution containing 2 mmol/L glucose (0‐10 min) before being exposed to 20 mmol/L glucose (10‐30 min). Islets were then exposed to 1 μmol/L fluoxetine in the continued presence of 20 mmol/L glucose for 20 minutes (30‐50 min; dashed lines) or to physiological salt solution containing 20 mmol/L glucose alone (30‐50 min; solid lines), with the final 10 minutes in the presence of 20 mmol/L glucose alone. The right panels show area under the curve data, n = 3‐4, *P < 0.05, **P < 0.01 versus control

Exposure of mouse and human islets to 0.1 and 1 μmol/L fluoxetine, either acutely (Figures 3A,B), or for 72 hours (Figures 3C,D), had no significant effect on basal insulin secretion at 2 mmol/L glucose concentration. Increasing the glucose concentration from 2 to 20 mmol/L significantly stimulated insulin secretion from mouse and human islets, and acute exposure to 1 μmol/L fluoxetine further potentiated glucose‐induced insulin secretion (Figure 3A and B). Similar results were observed following pre‐incubation of mouse (Figure 3C) and human (Figure 3D) islets to fluoxetine for 72 hours, where significantly increased glucose‐induced insulin secretion was observed after chronic exposure to 1 μmol/L fluoxetine.

Similar stimulatory effects of 1 μmol/L fluoxetine were observed in dynamic insulin secretion experiments, in which increasing the glucose concentration from 2 to 20 mmol/L initiated insulin secretory responses from mouse islets (Figure 3E), and from islets isolated from lean (BMI 20 kg/m^2^; Figure 3F) and obese (BMI 36 kg/m^2^; Figure 3G) donors. Acute exposure of mouse and human islets to 1 μmol/L fluoxetine led to significant potentiation of glucose‐induced insulin secretion that was rapid in onset and reversible upon removal of fluoxetine from the perifusion media (Figures 3E‐G, dashed lines).

### 3.4 Intermittent intraperitoneal administration of fluoxetine to *ob*/*ob* mice improves glucose tolerance in vivo, promotes beta‐cell proliferation and enhances insulin secretion ex vivo

**FIGURE 4:**
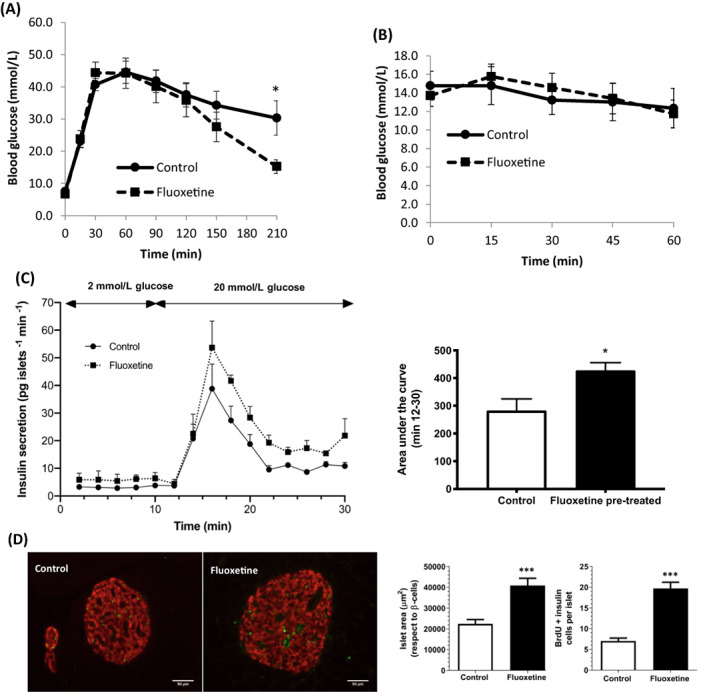
Effects of intermittent intraperitoneal administration of fluoxetine to *ob/ob* mice. Fluoxetine (10 mg/kg) or vehicle (DMSO) was administered intraperitoneally four times over the course of 14 days to 29‐week‐old *ob/ob* mice, then mice were subjected to intraperitoneal glucose (A, n = 5, *P < 0.05 vs. control) and insulin (B) tolerance tests after a single administration of glucose (2 g/kg body weight) or insulin (0.75 U/kg body weight) in the presence of fluoxetine or DMSO. Islets isolated from fluoxetine‐ and DMSO‐treated mice were perifused at 0.5 mL/min with a physiological salt solution containing 2 mmol/L glucose (0‐10 min) and 20 mmol/L glucose (10‐30 min). Insulin secretion from perifused islets was measured by radioimmunoassay (C, left panel, n = 3) and areas under the curve were calculated from the perifusion data (C, right panel, n = 3, *P < 0.05 vs. control). Wax‐embedded sections of pancreas from fluoxetine‐ and DMSO‐treated mice were immunostained with antibodies directed against 5‐bromo‐2′‐deoxyuridine (BrdU; green) and insulin (red; D, left panel, scale bars: 50 μm). Islet area and the number of BrdU‐positive beta cells per islet were quantified by analysis of multiple acquisitions of n = 99‐115 islets (D, right panel, ***P < 0.001 vs. control)

There were no significant differences (*P* > 0.8) in the body weight of the control mice (58.6 ± 2.2 g) or those treated with fluoxetine (57.9 ± 1.2 g), nor were there significant differences in the fasting glucose concentrations of control (7.5 ± 0.6 mmoL/L) and fluoxetine‐treated mice (6.7 ± 0.5 mmol/L; *P* > 0.3). Mice that had been administered four doses of 10 mg/kg fluoxetine over 14 days showed similar responses to glucose delivery to those of control mice over 120 minutes (Figure 4A). However, extending glucose measurement for a further 90 minutes revealed that, while control mice reached a plateau of 30.3 ± 5.4 mmol/L glucose 210 minutes after glucose administration, those that had received fluoxetine showed improved glucose clearance (15.2 ± 2.2 mmol/L at *t* = 210; *P* < 0.05). As expected, insulin tolerance testing indicated that the control *ob/ob* mice were overtly insulin‐resistant and those that had been pre‐exposed to fluoxetine did not show any improvements in insulin sensitivity (Figure 4B), suggesting that the improved glucose handling upon fluoxetine administration was a direct result of enhanced beta‐cell function and/or mass.

Islets isolated from mice treated with fluoxetine exhibited a more robust insulin secretory response to 20 mmol/L glucose than islets from control mice (Figure 4C). In addition, islet size was increased in mice treated with fluoxetine and there were more beta cells showing incorporation of BrdU (Figure 4D). Quantification of islet area in multiple sections indicated that it was significantly increased after exposure to fluoxetine and this was associated with significantly increased numbers of BrdU and insulin double‐positive cells. These data indicate that in vivo chronic delivery of fluoxetine promotes beta‐cell proliferation, which, together with direct effects of fluoxetine to potentiate insulin secretion, can overcome the intrinsic insulin resistance of *ob/ob* mice to marginally improve glucose tolerance.

### 3.5 Possible mechanisms of actions of fluoxetine in beta cells

**FIGURE 5:**
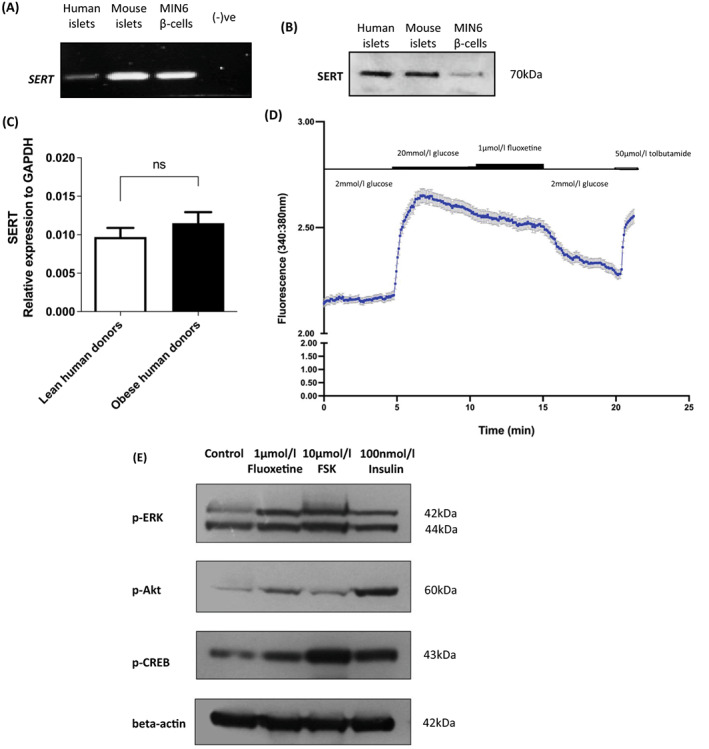
Possible mechanisms of actions of fluoxetine in beta cells. Products of PCR using cDNA from human islets, mouse islets and MIN6 beta cells, and species‐specific primers for SERT were electrophoretically separated on 1.8% agarose gels. Non‐reverse‐transcribed RNA was used as a negative control (A). 50 μg protein lysates from human islets, mouse islets and MIN6 beta cells were separated on a 10% polyacrylamide gel and SERT protein expression was detected by Western blot analysis (B). SERT mRNA expression was quantified in islets isolated from lean (body mass index [BMI] 21.0 ± 1.5 kg/m^2^) and obese (BMI 31.3 ± 0.3 kg/m^2^) donors and expressed relative to GAPDH mRNA (C). Real‐time changes in intracellular calcium in response to exposure to 20 mmol/L glucose, 1 μmol/L fluoxetine and 50 μmol/L tolbutamide were recorded in Fura‐2‐loaded dispersed mouse islet cells (D, mean ± SEM, n = 50 cells). MIN6 cells were incubated with 1 μmol/L fluoxetine or vehicle (0.005% v/v DMSO) for 24 hours before Western blotting for phosphorylation of ERK (p‐ERK), Akt (p‐Akt) and CREB (p‐CREB) using antibodies against the phosphorylated forms of the corresponding proteins. Levels of protein phosphorylation in response to fluoxetine were compared to those induced by 10‐minute exposure of MIN6 beta cells to 10 μmol/L forskolin or 100 nmol/L insulin. Expression of beta actin was used as loading control (E). Data are representative of two (for phospho‐Akt) or three (for phospho‐ERK and phospho‐CREB) different experiments

Using primers specific for human SERT, an amplicon of 187 bp was obtained when human islet cDNA was used as a template, and a 207‐bp product was detected after amplification of mouse islet and MIN6 beta‐cell cDNA using primers for mouse SERT (Figure 5A). Additionally, immunoreactive proteins of 70 kDa were detected in Western blots of human and mouse islets, and MIN6 beta cells, using an antibody directed against both human and mouse SERT (Figure 5B). These observations confirm that SERT is expressed by islets and are consistent with fluoxetine acting on beta cells to inhibit SERT and maintain local endogenous 5‐HT levels. Quantification of SERT expression in islets from lean and obese donors indicated that there were no significant differences in the expression levels of SERT mRNA in islets during obesity (Figure 5C).

Since elevations in [Ca^2+^]_i_ are important in the stimulation of insulin secretion, the effects of fluoxetine were evaluated in Fura‐2‐loaded mouse islet cells using single‐cell calcium microfluorimetry. Results from these experiments indicated that, as expected, exposure to 20 mmol/L glucose resulted in a rapid and sustained elevation in [Ca^2+^]_i_ and this was not further enhanced by the addition of 1 μmol/L fluoxetine (Figure 5D). Tolbutamide, the ATP‐sensitive K^+^ channel blocker, stimulated increases in [Ca^2+^]_I_ following exposure to fluoxetine, demonstrating maintenance of the membrane potential and capacity of the beta cells to elevate calcium.

As ERK2, CREB and Akt are known to have pro‐proliferative and anti‐apoptotic effects in a range of cell types, including beta cells,^28^, ^31^, ^32^, ^33^ the ability of fluoxetine to phosphorylate and thus activate these intracellular mediators was examined. It can be seen from Figure 5E that incubation of MIN6 beta cells with 1 μmol/L fluoxetine resulted in enhanced phosphorylation of ERK2, Akt and CREB. Phosphorylation of these proteins was also stimulated by exposure of MIN6 beta cells to 10 μmol/L forskolin or 100 nmol/L insulin.

## 4 DISCUSSION

Fluoxetine (Prozac®) is a safe and effective SSRI that is used as an antidepressant by millions of people worldwide. Several independent studies have indicated beneficial effects of fluoxetine on HbA1c levels^11^, ^12^, ^13^, ^14^, ^34^, ^35^ and the data presented here are consistent with a glucose‐lowering effect of fluoxetine that is mediated, at least in part, through promoting beta‐cell mass expansion and potentiating glucose‐stimulated insulin secretion.

Previous reports of inhibition of insulin secretion from MIN6 beta cells by fluoxetine, resulting from oxidative stress and impaired mitochondrial bioenergetics^22^ and reduction in endoplasmic reticulum calcium storage,^23^ are in contrast to our observations of enhanced insulin secretion from both mouse and human islets. These earlier studies used fluoxetine at concentrations of between 10 μmol/L and 100 μmol/L, which are considerably higher than its therapeutically relevant steady‐state plasma concentrations of <3 μmol/L.^25^, ^36^, ^37^ In vitro fluoxetine use at excessive concentrations may hinder its bona fide effects. Indeed, our observations that 10 μmol/L fluoxetine induced cytotoxic effects on beta cells whereas therapeutic fluoxetine concentrations (0.1 and 1 μmol/L) did not compromise beta‐cell viability support this notion and prompted us to re‐evaluate the direct effects of therapeutically relevant concentrations of fluoxetine on beta‐cell function and mass.

We have demonstrated here, for the first time, that when used at the therapeutically relevant concentration of 1 μmol/L, fluoxetine potentiated glucose‐induced insulin secretion from mouse and human islets in vitro, indicating its direct stimulatory effect on the endocrine pancreas. We used islets from obese as well as lean donors as there is evidence that a large proportion of people taking SSRIs are overweight or obese.^38^, ^39^, ^40^ Our data indicated that 1 μmol/L fluoxetine did potentiate glucose‐induced insulin secretion from islets obtained from the obese donor, suggesting that obese individuals taking this SSRI would benefit from its direct effects to enhance insulin secretion and the lack of effect of fluoxetine on basal insulin secretion at 2 mmol/L glucose suggests that it is unlikely to induce hypoglycaemia in the fasting state. We also found that 0.1 and 1 μmol/L fluoxetine promoted beta‐cell proliferation and protected islet cells from cytokine‐induced apoptosis. These in vitro observations are consistent with the improved glucose tolerance induced by fluoxetine administration to *ob/ob* mice, a leptin‐deficient model of obesity and hyperglycaemia. The improved glycaemic control was not associated with any reduction in insulin resistance in this model, suggesting that the primary site of action of fluoxetine was the beta cells. Consistent with this, islets isolated from fluoxetine‐treated mice demonstrated increased capacity to secrete insulin in response to 20 mmol/L glucose. Furthermore, measurement of BrdU incorporation into islet cells from the fluoxetine‐treated mice showed significantly elevated levels of beta‐cell proliferation. Taken together, it is possible that the increased insulin secretory capacity and augmented beta‐cell mass account for the improvement in glucose tolerance seen in *ob/ob* mice chronically administered fluoxetine. Nonetheless, the overt insulin resistance of these mice most likely accounted for the delayed reduction in glucose levels observed in the ipGTTs, and use of milder mouse models of glucose dysregulation may allow the in vivo glucose‐lowering effect of fluoxetine to be more readily seen. The dose and duration of fluoxetine used in the in vivo experiments are in line with previously published studies where fluoxetine has been delivered in vivo^41^, ^42^, ^43^ and plasma concentrations of fluoxetine remain in the low micromolar range even upon chronic use at 10 mg/kg/d.^44^, ^45^

We detected SERT mRNA and protein in MIN6 beta cells and in mouse and human islets, and exposure of human islets to another SSRI, fluvoxamine, significantly increased the level of extracellular 5‐HT.^46^ Although it has been reported that SERT availability is decreased in brain regions in obesity^47^ we found that islet expression of SERT mRNA was not changed significantly during obesity, when there is an increased metabolic demand for insulin. Our observations provide initial evidence that fluoxetine inhibition of beta‐cell SERT may maintain local levels of endogenous 5‐HT in the islet extracellular space, allowing it to stimulate insulin secretion.^15^, ^19^, ^20^, ^48^, ^49^ These 5‐HT‐dependent effects may be mediated through specific members of the 5‐HT receptor superfamily. Islets express several 5‐HT receptors, among them both the G_αq_‐coupled 5‐HT_2_ and the G_s_‐coupled 5‐HT_4_ have been reported to promote insulin exocytosis.^48^, ^49^ However, we did not observe increases in [Ca^2+^]_i_ when mouse islet cells were exposed to fluoxetine, suggesting that its ability to potentiate insulin secretion is independent of [Ca^2+^]_i_ influx or mobilization and therefore G_αq_‐coupled 5‐HT_2_ signalling is unlikely to be important in its effects to stimulate insulin secretion. The 5‐HT‐dependent effects of fluoxetine could be mediated through mechanisms other than activation of 5‐HT receptors. For example, it has been proposed that 5‐HT may be covalently coupled to the small GTPases Rab3a and R2b27a in a process termed serotonylation, and this can lead to increased insulin secretion.^18^

There are several mechanisms through which fluoxetine could promote beta‐cell mass expansion. Thus, increased local 5‐HT after exposure of islets to fluoxetine could play a role in driving beta‐cell proliferation^15^, ^19^, ^20^, ^21^ and this is consistent with fluoxetine‐induced proliferation of cells in the central nervous system being mediated through 5‐HT‐dependent modulation of 5‐HT_1_ and/or 5‐HT_2._ ^50^ Activation of the transcription factor nuclear factor κB (NFκB) by proinflammatory cytokines such as IL‐1β and TNFα promotes beta‐cell apoptosis^51^, ^52^ and fluoxetine has previously been reported to inhibit NFkB signalling in other cell types,^53^ so it is possible that reduction in NF‐kB expression or activity may be involved in fluoxetine‐mediated protection of cytokine‐induced beta‐cell apoptosis. The increased insulin secretion in response to fluoxetine could also be responsible for its proliferative and anti‐apoptotic effects since insulin itself has an autocrine effect to promote beta‐cell proliferation and reduce beta‐cell apoptosis.^54^ Alternatively, the increase in beta‐cell proliferation could be a consequence of increased phosphorylation of the transcription factor CREB, which can be induced directly by fluoxetine, or through 5‐HT‐dependent or ‐independent activation of CaMK4 and/or ERK2 (p42 MAPK) phosphorylation.^41^, ^55^, ^56^, ^57^ We, and others, have identified that ERK2 plays important roles in regulating beta‐cell proliferation^32^, ^33^, ^58^ and we have previously demonstrated that CaMK4‐mediated CREB phosphorylation regulates Irs2 expression, and subsequently beta‐cell proliferation and apoptosis.^59^ Consistent with this, we found that 1 μmol/L fluoxetine increased phosphorylation of ERK2 and CREB in MIN6 beta cells. Fluoxetine also stimulated Akt phosphorylation, as it is reported to do in neural stem cells,^60^ and this may contribute to its protection against cytokine‐induced apoptosis.^61^

In summary, data presented in this study are consistent with a direct role for fluoxetine in promoting beta‐cell secretory capacity and maintaining functional beta‐cell mass. These effects could contribute to the established satiety‐inducing effects of elevated 5‐HT to mediate the improved glucose homeostasis observed in fluoxetine‐treated patients with depression and T2D. Results presented here may pave the way for fluoxetine to be recommended as a preferential antidepressant for patients with concomitant occurrence of depression and diabetes.

### PEER REVIEW

The peer review history for this article is available at https://publons.com/publon/10.1111/dom.14791.

## Data Availability

The data that support the findings of this study are available from the corresponding author upon reasonable request.
